# Cannabis and tobacco use among adolescents aged 12-17 years from 16 low- and middle-income countries

**DOI:** 10.7189/jogh.13.04066

**Published:** 2023-07-27

**Authors:** Qian Wang, Hui Wang

**Affiliations:** 1 School of Public Health, Shanghai Jiao Tong University School of Medicine, Shanghai, China; 2 Key Laboratory of Systems Medicine for Cancer, Center for Single-Cell Omics, School of Public Health, Shanghai Jiao Tong University School of Medicine, Shanghai, China

## Abstract

**Background:**

We aimed to examine the association between cannabis use and tobacco use in 12-17-year-old adolescents residing in low- and middle-income countries (LMICs).

**Methods:**

We used data from the Global School-based Student Health Survey collected between 2012 and 2022. We selected 16 LMICs based on pre-defined inclusion criteria and compared the prevalence of cannabis use for female and for male adolescents for each country. We estimated age- and sex-standardised distributions of tobacco use, school truancy, suicide attempt, sex with multiple partners, physical fighting, perceived school kindness, and parental monitoring were estimated. We used multivariable logistic regression to estimate odds ratios (ORs) and 95% confidence intervals (CIs) measuring associations between cannabis use and other variables. We generated pooled estimates through random effects meta-analyses.

**Results:**

The overall pooled prevalence of cannabis use was 4.3% (95% CI = 3.2-5.9), with significant between-country heterogeneities (*I*^2^ = 91.4%; *P* = 0.000). Cannabis use was more prevalent in males than in females in each country. Tobacco use was strongly associated with cannabis use in all 16 LMICs. Tobacco users had approximately 11 to 14 times greater odds of cannabis use than non-tobacco users. Sex with multiple partners, suicide attempts, and school truancy had a robust association with cannabis use in over half of LMICs.

**Conclusions:**

There is a need for comprehensive preventive measures targeting multiple risk behaviours associated with cannabis use for adolescents in LMICs.

*Cannabis sativa*, commonly known as marijuana, has been cultivated for its industrial, medicinal, and psychotropic properties for centuries. Its primary psychoactive compound – delta-9-tetrahydrocannabinol (THC) – produces effects such as euphoria, relaxation, and changes in perceptions [1]. According to the 2021 United Nations Office on Drugs and Crime (UNODC) World Drug Report, cannabis was the most commonly cultivated, trafficked, and used drug globally [2]. Between 2010 and 2019, past-year cannabis use increased by 18% worldwide [2]. In 2019, cannabis was the most commonly used drug among youth aged 15-16 years, especially among those from economically prosperous countries in Oceania (Australia and New Zealand), Americas (North America), and Europe (Western and Central Europe) [2].

Although adolescents exhibit overall good health, their brains are still developing complex neural networks and architecture for undertaking higher-order cognitive and emotional capacities, making them more vulnerable than adults to consequences of cannabis use [3]. According to a 2017 report by the National Academies of Sciences, Engineering, and Medicine, there is substantial evidence that initiating cannabis use at a younger age increases the odds of developing cannabis use disorders later in life; furthermore, cannabis use during adolescence is associated with lower academic achievement, employment, income, and impairments in social relationships and roles [4]. There is also evidence that cannabis use can increase the risk of developing schizophrenia, other psychoses, and even suicidal thoughts [4]. Hence, cannabis use during adolescence might have negative repercussions on short- and long-term health.

There seems to be a strong relationship between use of tobacco and cannabis in youth residing in developed countries, as “mixing” (adding loose-leaf tobacco to cannabis in joints), “blunt smoking” (adding cannabis to hollowed-out cigars/cigarillos), and “chasing” (using tobacco immediately following cannabis use or vice versa) have gained in popularity. Systematic reviews suggest that there may be genetic and environmental underpinnings of cannabis and tobacco co-use [5,6] For example, genetic predispositions to impulsivity, sensation-seeking, and disinhibition may contribute to initiation of both cannabis and tobacco use; because inhalation is the most common route of administration for both products, it is possible that using one substance would facilitate use of the other [5,6]. Compared with using either substance alone, co-use of cannabis and tobacco is associated with elevated risks of respiratory distress, cannabis use disorders, nicotine dependence, psychosocial problems, and poorer cannabis or tobacco cessation outcomes [5-7]. Hence, for developing adolescents, preventing or reducing co-use of cannabis and tobacco has important public health implications.

Older studies found that, youth cannabis use was more widespread in developed countries than in less developed countries [8]. However, a plateau in use was observed in a few developed countries since 2000 [9]. A study using data from the 2002-2010 Health Behavior in School-aged Children (HBSC) survey found that, among 30 European and North American countries, cannabis use decreased over time in more affluent countries up to 2010, but appeared to stabilise or even increase in developing countries across Europe [10]. The 2021 UNODC World Drug Report similarly found that cannabis use among young people declined or stabilised in some developed countries (mainly Western and Central Europe, North America, Australia and New Zealand) by 2010, but increased in many countries in Africa and Asia after 2010 [2]. Simultaneously, the largest decreases in the number of smokers between 1990 and 2019 were observed in certain high-income regions (mainly North America, Western Europe, Australia and New Zealand), Latin America, and the Caribbean region, which mostly consist of upper-middle income countries [11], while the largest increases were observed in the Middle East and North Africa region (MENA), and sub-Saharan Africa, which mostly consist of low- and middle-income countries (LMICs) [11].

Contrary to tobacco use, there is a general lack of population-based data on cannabis use and associated risk factors or outcomes in LMICs. The Global School-based Student Health Survey (GSHS) is one of the few that collect data on health behaviours among students aged 12-17 years worldwide, especially those from LMICs. Since 2009, the GSHS has included two items to assess cannabis use, one on frequency of lifetime usage and the other on frequency of past 30-day usage. No study has focused exclusively on the association between cannabis and tobacco use among adolescents from LMICs on a population level. Given the strong correlation between tobacco and cannabis use generally found in adolescents from more prosperous regions, we aimed to examine this association in adolescents from LMICs at the population level.

## METHODS

### Sample

We used data collected between 2012 and 2022 by the GSHS, jointly developed by the USA Centers for Disease Control and Prevention (CDC), the World Health Organization (WHO), and related agencies. We included countries categorised as LMICs per the World Bank income classification in the corresponding year when the most recent survey was conducted, and where data were collected and available in public use files for adolescents aged 12-17 years, data for frequency of past 30-day cannabis use, tobacco use, and covariates (age, gender, food security, school truancy, sex with multiple partners, physical fighting, suicide attempt, perceived school kindness, and parental monitoring).

### Cannabis use

We assessed cannabis use based on responses to the item “During the past 30 days, how many times have you used marijuana?”. Response options included “0 times”, “1 or 2 times”, “3 to 9 times”, “10 to 19 times”, and “30 or more times”, which we then dichotomised into “no” (“0 times”), and “yes” (“1 or 2 times” or more).

### Tobacco use

We assessed tobacco use based on combinations of responses to two items: “During the past 30 days, on how many days did you smoke cigarettes?” and “During the past 30 days, on how many days did you use any tobacco products other than cigarettes?” Response options for both questions were “0 days”, “1 or 2 days”, “3 to 5 days”, “6 to 9 days”, “10 to 19 days”, “20 to 29 days”, and “All 30 days”. We also dichotomised the responses as “no” (“0 days” for both items), and “yes” (at least “1 or 2 days” for either item).

### Covariates

Covariates were sociodemographic and key determinant factors of cannabis use. The former included age (12-17 years), gender (females vs males), food security (during the past 30 days, those went hungry because there was not enough food at home most of the time or always vs never, rarely, or sometimes). Based on findings from previous studies [12-16], we considered the following factors as main determinants of cannabis use: school truancy (missed classes or school without permission on zero days vs one or more days during the past 30 days), sex with multiple partners (never had or had sexual intercourse with one person vs with two or more persons during lifetime), physical fighting (were in a physical fight zero times vs one or more times during the past 12 months), suicide attempt (attempted suicide zero times vs one or more times during the past 12 months), perceived school kindness (most of students in school were kind and helpful most of the time or always vs never, rarely, or sometimes), and level of parental monitoring (parents or guardians really know what you were doing with your free time most of the time or always vs never, rarely, or sometimes).

### Statistical analysis

We analysed the data using STATA (version 15.1, StataCorp LP, College Station, Texas). We imputed missing values using multiple imputations by chained equations (MICE), which can handle mixed types of variables, including binary, categorical, or continuous ones. It imputes multivariate data by running a series of regression models, one for each variable with missing data, with each variable conditionally modelled upon other ones in the data. We included all variables used in data analysis to set up the imputation model, generating 10 imputed data sets (M = 10) on which we compared the prevalence of past 30-day cannabis use for female and male adolescents in each country. We estimated age- and sex-standardised distributions of other variables and measured their association with cannabis use by performing multivariable logistic regression to estimate odds ratios (ORs) and 95% confidence intervals (CIs). We generated regional and overall pooled estimates using random effects meta-analyses.

## RESULTS

We included 50 488 adolescent aged 12-17 years from 16 LMICs from four WHO regions: the African region (n = 6), Southeast Asia (n = 4), Region of the Americas (n = 3), and Western Pacific region (n = 3) (**Table 1**).

**Table 1:** Country characteristics, GSHS, 2013-2017

Countries by region	Income group	Survey year	Sample size	Male%
**African region**				
Benin	Low-income	2016	1584	68.7
Liberia	Low-income	2017	1261	51.5
Mozambique	Low-income	2015	1320	52.0
Tanzania	Low-income	2014	3434	49.0
Mauritius	Upper middle-income	2017	2970	46.8
Namibia	Upper middle-income	2013	3347	44.7
**Southeast Asia**				
Nepal	Low-income	2015	6240	48.8
Indonesia	Lower middle-income	2015	10695	48.9
Timor Leste	Lower middle-income	2015	3006	48.1
Thailand	Upper middle-income	2015	5630	46.8
**Region of the Americas**				
The Dominican Republic	Upper middle-income	2016	1359	50.4
Jamaica	Upper middle-income	2017	1608	48.7
Suriname	Upper middle-income	2016	1903	49.2
**Western Pacific**				
Vanuatu	Lower middle-income	2016	2042	49.4
Fiji	Upper middle-income	2016	3054	49.6
Wallis and Futuna	Upper middle-income	2015	1035	49.1

GSHS – Global School-based Student Health Survey

**Figure 1:**
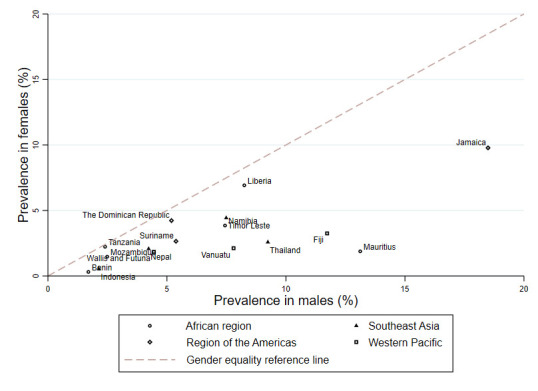
Prevalence of past 30-day cannabis use among male and female adolescents aged 12-17 years from across 16 LMICs, GSHS, 2013-2017.

**Table 2** presents age- and sex-adjusted prevalence of past 30-day cannabis use. The pooled prevalence in all countries was 4.3% (95% CI = 3.2-5.9). Regional pooled prevalence was higher in Americas (6.6%; 95% CI = 2.6-16.6) and Western Pacific (4.7; 95% CI = 3.1-7.2), and lower in Southeast Asia (3.5%; 95% CI = 1.9-6.5) and Africa (3.8%; 95% CI = 2.4-6.1). We found a high level of between-country heterogeneity within all four regions (*P* = 0.000). Country-specific prevalence of past 30-day cannabis use was highest in Jamaica (14.0%; 95% CI = 10.9-17.2) and Liberia (7.6%; 95% CI = 5.0-10.3). Of all countries, Benin (1.3%; 95% CI = 0.4-2.2) and Indonesia (1.3%; 95% CI = 0.8-1.9) had the lowest prevalence of past 30-day cannabis use. Cannabis use was more prevalent in male than in female adolescents in all countries (**Figure 1**).

**Table 2:** Age- and sex-adjusted prevalence of sample characteristics, GSHS, 2013-2017

Countries by region	Cannabis use, % (95% CI)	Tobacco use, % (95% CI)	School truancy, % (95% CI)	Suicide attempt, % (95% CI)	Sex with multiple partners, % (95% CI)	Physical fighting, % (95% CI)	Perceived school kindness, % (95% CI)	Parental monitoring, % (95% CI)
**African Region**								
Benin	1.3 (0.4-2.2)	7.4 (5.2-9.5)	21.1 (17.7-24.5)	13.2 (10.3, 16.0)	22.5 (18.8-26.1)	28.0 (24.2-31.8)	22.5 (19.0-26.1)	34.1 (30.7-37.6)
Liberia	7.6 (5.0-10.3)	13.5 (10.0-17.0)	43.8 (39.7-47.9)	29.0 (25.2, 32.9)	16.3 (13.4-19.2)	42.0 (37.0-46.9)	37.9 (34.3-41.4)	36.8 (33.6-40.0)
Mozambique	2.0 (0.8-3.1)	5.0 (2.4-7.7)	23.3 (16.8-29.7)	17.7 (13.7, 21.7)	19.5 (15.2-23.7)	36.3 (32.0-40.7)	34.1 (27.4-40.8)	40.4 (32.8-48.0)
Tanzania	2.3 (1.4-3.2)	6.7 (5.0-8.4)	27.7 (23.8-31.7)	10.7 (8.4, 13.0)	4.9 (4.0-5.9)	29.3 (26.1-32.4)	36.1 (32.5-39.7)	39.2 (35.7-42.6)
Mauritius	7.1 (5.3-8.9)	20.1 (17.8-22.4)	24.8 (21.0-28.7)	12.9 (10.8, 15.0)	7.7 (5.9-9.6)	28.7 (25.7-31.8)	35.9 (32.0-39.9)	44.4 (41.6-47.2)
Namibia	5.5 (3.9-7.1)	12.8 (10.9-14.8)	25.1 (22.1-28.1)	25.9 (22.1, 29.7)	20.2 (18.3-22.0)	35.1 (31.4-38.8)	27.1 (24.6-29.6)	34.7 (32.5-36.9)
**Pooled estimate**	3.8 (2.4-6.1)	10.1 (6.8-15.0)	27.0 (20.8-34.9)	17.1 (12.1, 24.2)	13.3 (8.6-20.8)	32.9 (29.0-37.3)	31.8 (27.3-37.2)	38.0 (34.5-41.9)
	*I*^2^ = 88.4%, *P* = 0.000	*I*^2^ = 95.0%, *P* = 0.000	*I*^2^ = 94.9%, *P* = 0.000	*I*^2^ = 95.6%, *P* = 0.000	*I*^2^ = 97.6%, *P* = 0.000	*I*^2^ = 86.1%, *P* = 0.000	*I*^2^ = 90.6%, *P* = 0.000	*I*^2^ = 86.3%, *P* = 0.000
**Southeast Asia**								
Nepal	3.1 (2.0-4.2)	7.9 (6.6-9.7)	28.4 (24.6-31.9)	10.3 (7.8-12.9)	3.8 (2.0-5.5)	39.2 (36.3-42.2)	53.5 (49.6-57.4)	49.9 (45.2-54.5)
Indonesia	1.3 (0.8-1.9)	12.5 (10.6-14.3)	19.9 (17.7-22.1)	3.8 (3.1-4.6)	1.3 (0.9-1.8)	23.4 (21.3-25.5)	39.3 (37.3-41.3)	39.8 (37.9-41.8)
Timor Leste	6.0 (4.6-7.4)	27.9 (25.8-30.0)	35.1 (32.1-38.1)	9.6 (7.4-11.9)	6.6 (5.0-8.1)	29.4 (26.8-32.1)	27.2 (24.3-30.1)	23.2 (20.9-25.5)
Thailand	5.8 (4.1-7.6)	14.0 (10.3-17.7)	20.4 (18.2-22.6)	13.2 (10.2-16.1)	7.4 (6.2-8.7)	25.8 (23.1-28.5)	39.3 (35.9-42.7)	42.5 (39.6-45.3)
Pooled estimate	3.5 (1.9-6.5)	14.0 (7.5-26.3)	25.2 (18.8-33.8)	8.4 (4.7-15.0)	4.0 (2.0-8.3)	28.9 (22.8-36.7)	38.8 (30.9-48.8)	37.4 (29.2-48.0)
	*I*^2^ = 93.1%, *P* = 0.000	*I*^2^ = 98.5%, *P* = 0.000	*I*^2^ = 96.7%, *P* = 0.000	*I*^2^ = 96.0%, *P* = 0.000	*I*^2^ = 96.3%, *P* = 0.000	*I*^2^ = 96.5%, *P* = 0.000	*I*^2^ = 97.3%, *P* = 0.000	*I*^2^ = 97.8%, *P* = 0.000
**Region of the Americas**								
The Dominican Republic	4.7 (2.2-7.3)	11.9 (7.4-16.3)	25.6 (22.0-29.1)	16.7 (12.5-20.9)	20.5 (14.5-26.4)	23.8 (19.5-28.1)	48.9 (42.8-55.0)	56.6 (51.7-61.4)
Jamaica	14.0 (10.9-17.2)	19.7 (16.4-23.1)	28.2 (24.4-31.9)	18.1 (15.2-21.0)	30.7 (27.8-33.6)	31.3 (27.7-34.8)	18.3 (14.9-21.7)	39.5 (36.5-42.5)
Suriname	3.9 (1.8-6.1)	14.4 (11.0-17.9)	21.4 (18.4-24.5)	11.3 (8.5-14.1)	12.5 (10.2-14.7)	21.7 (18.9-24.6)	51.0 (47.0-55.0)	46.6 (42.4-50.8)
Pooled estimate	6.6 (2.6-16.6)	15.6 (11.6-20.8)	25.0 (21.3-29.2)	15.2 (11.5-20.1)	19.9 (10.6-37.3)	25.4 (19.9-32.4)	36.0 (21.8-59.5)	47.0 (38.1-43.7)
	*I*^2^ = 91.2%, *P* = 0.000	*I*^2^ = 74.3%, *P* = 0.020	*I*^2^ = 73.5%, *P* = 0.023	*I*^2^ = 79.5%, *P* = 0.008	*I*^2^ = 97.3%, *P* = 0.000	*I*^2^ = 88.9%, *P* = 0.000	*I*^2^ = 98.0%, *P* = 0.000	*I*^2^ = 94.7%, *P* = 0.000
**Western Pacific**								
Vanuatu	4.9 (3.6-6.2)	24.9 (21.9-28.0)	55.4 (51.1-59.7)	22.6 (19.5-25.7)	8.6 (6.8-10.3)	40.8 (37.0, 44.5)	37.2 (33.0-41.5)	22.5 (20.3-24.6)
Fiji	7.4 (3.9-11.0)	15.4 (10.6-20.3)	23.8 (20.5-27.1)	11.3 (8.6-13.9)	7.8 (5.4-10.2)	32.9 (29.1-36.6)	50.6 (47.5-53.8)	49.1 (45.0-53.2)
Wallis and Futuna	3.1 (2.0-4.3)	31.5 (27.4-35.6)	28.8 (25.4-32.3)	14.7 (12.3-17.1)	15.1 (12.8-17.4)	33.9 (30.8-36.9)	61.6 (57.6-65.6)	45.5 (42.0-49.0)
Pooled estimate	4.7 (3.1-7.2)	23.9 (17.9-32.0)	33.7 (19.4-58.5)	15.7 (10.6-23.3)	10.2 (6.5-15.9)	35.7 (31.2-40.9)	49.0 (38.6-62.2)	36.9 (23.4-58.2)
	*I*^2^ = 73.5%, *P* = 0.023	*I*^2^ = 89.0%, *P* = 0.000	*I*^2^ = 98.7%, *P* = 0.000	*I*^2^ = 93.4%, *P* = 0.000	*I*^2^ = 92.2%, *P* = 0.000	*I*^2^ = 82.2%, *P* = 0.004	*I*^2^ = 96.6%, *P* = 0.000	*I*^2^ = 98.8%, *P* = 0.000
**Total pooled estimate**	4.3 (3.2-5.9)	14.0 (11.0-17.7)	27.2 (22.9-32.4)	13.8 (10.7-17.6)	10.2 (7.3-14.2)	30.9 (28.0-34.0)	37.2 (32.5-42.7)	39.2 (35.3-43.7)
	*I*^2^ = 91.4%, *P* = 0.000	*I*^2^ = 96.7%, *P* = 0.000	*I*^2^ = 97.0%, *P* = 0.000	*I*^2^ = 96.3%, *P* = 0.000	*I*^2^ = 98.2%, *P* = 0.000	*I*^2^ = 92.5%, *P* = 0.000	*I*^2^ = 97.3%, *P* = 0.000	*I*^2^ = 96.5%, *P* = 0.000

CI – confidence interval, GSHS – Global School-based Student Health Survey

**Figure 2:**
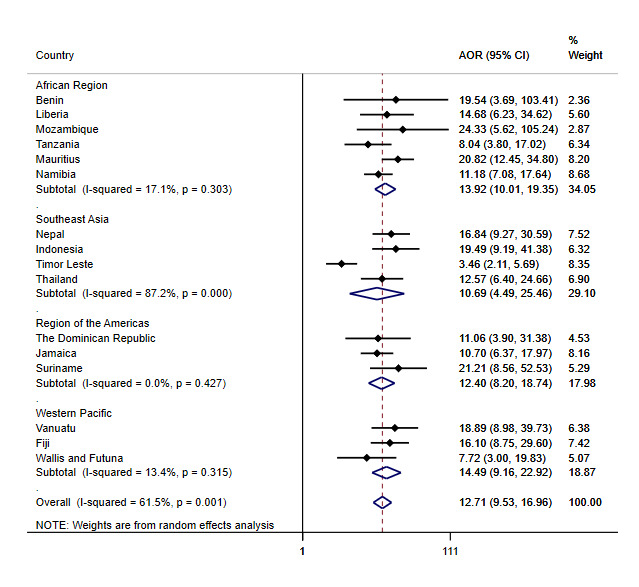
Adjusted association between past 30-day cannabis use and any tobacco use among adolescents aged 12-17 years from across 16 LMICs, GSHS, 2013-2017. AOR – adjusted odds ratio, CI – confidence interval.

Tobacco use was independently and strongly associated with cannabis use in all 16 countries (**Table 3** and **Figure 2**). Regional pooled estimates indicate that, the association was significant in all four regions, and the level of between-country heterogeneity was low (*I*^2^ between 0% and 20%) in all regions except for in Southeast Asia (*I*^2^ = 87.2%; *P* = 0.000). The odds of cannabis use were approximately 11 to 14 times greater in tobacco users than in non-tobacco users.

**Table 3:** Country-wise and regional pooled associations between past 30-d cannabis use and predictor variables among adolescents aged 12-17 y from across 16 LMICs, GSHS 2013-2017

Countries by region	Age, AOR (95% CI)	Sex (vs male), AOR (95% CI)	Food insecurity, AOR (95% CI)	Suicide attempt, % (95% CI)	Tobacco use, AOR (95% CI)	School truancy, AOR (95% CI)	Suicide attempt, AOR (95% CI)	Sex with multiple partners, AOR (95% CI)	Physical fighting, AOR (95% CI)	Perceived school kindness, AOR (95% CI)	Parental monitoring, AOR (95% CI)
**African Region**											
Benin	1.82 (0.95-3.52)	0.42 (0.11-1.68)	0.54 (0.19-1.59)	19.54 (3.69-103.41)†	4.90 (1.10-21.81)*	0.42 (0.11-1.68)	11.47 (2.09-62.96)†	2.27 (0.54-9.52)	0.81 (0.20-3.29)	0.44 (0.07-2.67)	0.21 (0.07-0.65)†
Liberia	1.10 (0.83-1.46)	0.76 (0.39-1.48)	2.62 (1.03-6.62)*	14.68 (6.23-34.62)‡	1.58 (0.63-3.99)	0.76 (0.39-1.48)	1.57 (0.73-3.40)	4.81 (2.37-9.73)‡	1.84 (0.76-4.46)	0.54 (0.24-1.24)	0.93 (0.43-2.03)
Mozambique	1.24 (0.69-2.23)	0.41 (0.03-5.98)	0.98 (0.14-6.81)	24.33 (5.62-105.24)†	4.09 (0.30-55.31)	0.41 (0.03-5.98)	3.26 (0.71-15.02)	2.15 (0.27-17.30)	3.74 (0.80-17.36)	1.46 (0.23-9.45)	2.28 (0.55-9.45)
Tanzania	0.79 (0.62-1.00)	0.96 (0.50-1.86)	1.27 (0.44-3.71)	8.04 (3.80-17.02)‡	2.61 (1.26-5.40)*	0.96 (0.50-1.86)	3.68 (1.70-7.96)†	3.26 (1.36-7.80)*	2.95 (1.30-6.70)*	0.77 (0.34-1.75)	0.48 (0.24-0.97)*
Mauritius	1.25 (1.03-1.52)*	0.25 (0.14-0.45)‡	0.52 (0.21-1.28)	20.82 (12.45-34.80)‡	2.17 (1.35-3.49)†	0.25 (0.14-0.45)‡	1.26 (0.59-2.67)	4.26 (2.41-7.54)‡	1.32 (0.74-2.34)	0.92 (0.52-1.62)	0.84 (0.46-1.54)
Namibia	1.04 (0.87-1.23)	0.93 (0.55-1.57)	1.79 (1.14-2.82)*	11.18 (7.08-17.64)‡	2.70 (1.62-4.50)†	0.93 (0.55-1.57)	1.85 (1.13-3.03)*	2.19 (1.41-3.39)†	1.54 (0.98-2.42)	0.88 (0.60-1.29)	0.76 (0.48-1.22)
Pooled estimate	1.08 (0.91-1.29)	0.60 (0.35-1.04)	1.17 (0.67-2.03)	13.92 (10.01-19.35)‡	2.41 (1.81-3.23)‡	0.60 (0.35-1.04)	2.21 (1.40-3.50)†	3.11 (2.29-4.22)‡	1.65 (1.23-2.21)†	0.83 (0.63-1.08)	0.70 (0.46-1.06)
	*I*^2^ = 56.7%, *P* = 0.041	*I*^2^ = 64.4%, *P* = 0.015	*I*^2^ = 53.3%, *P* = 0.058	*I*^2^ = 17.1%, *P* = 0.303	*I*^2^ = 0.0%, *P* = 0.813	*I*^2^ = 64.4%, *P* = 0.015	*I*^2^ = 42.6%, *P* = 0.121	*I*^2^ = 7.4%, *P* = 0.369	*I*^2^ = 0.0%, *P* = 0.449	*I*^2^ = 0.0%, *P* = 0.832	*I*^2^ = 45.8%, *P* = 0.100
**Southeast Asia**											
Nepal	0.94 (0.77-1.14)	0.62 (0.37-1.05)	2.46 (0.98-6.17)	16.84 (9.27-30.59)‡	3.50 (2.03-6.04)‡	0.62 (0.37-1.05)	1.37 (0.70-2.68)	5.07 (2.57-10.00)‡	1.37 (0.76-1.47)	0.66 (0.41-1.06)	0.72 (0.34-1.52)
Indonesia	0.64 (0.49-0.83)†	0.90 (0.46-1.78)	1.25 (0.48-3.27)	19.49 (9.19-41.38)‡	2.05 (1.24-3.37)†	0.90 (0.46-1.78)	3.68 (1.92-7.08)‡	19.62 (8.67-44.40)‡	1.34 (0.74-2.45)	0.39 (0.21-0.73)†	0.55 (0.25-1.21)
Timor Leste	0.78 (0.70-0.87)‡	1.16 (0.70-1.92)	2.02 (1.23-3.33)†	3.46 (2.11-5.69)‡	2.96 (1.69-5.18)†	1.16 (0.70-1.92)	4.06 (2.42-6.84)‡	5.49 (2.61-11.54)†	2.06 (1.33-3.19)†	0.94 (0.62-1.43)	1.04 (0.68-1.59)
Thailand	0.92 (0.82-1.05)	0.73 (0.40-1.34)	1.65 (0.69-3.96)	12.57 (6.40-24.66)‡	2.98 (1.90-4.66)‡	0.73 (0.40-1.34)	1.74 (0.90-3.36)	6.43 (3.78-10.92)‡	2.46 (1.62-3.74)‡	0.52 (0.28-0.97)*	0.55 (0.26-1.17)
Pooled estimate	0.83 (0.72-0.95)†	0.83 (0.63-1.11)	1.88 (1.31-2.70)†	10.69 (4.49-25.46)‡	2.80 (2.17-3.61)‡	0.83 (0.63-1.11)	2.50 (1.46-4.26)†	7.41 (4.32-12.71)‡	1.76 (1.30-2.38)‡	0.63 (0.43-0.91)*	0.78 (0.56-1.09)
	*I*^2^ = 67.3%, *P* = 0.027	*I*^2^ = 3.8%, *P* = 0.373	*I*^2^ = 0.0%, *P* = 0.756	*I*^2^ = 87.2%, *P* = 0.000	*I*^2^ = 0.0%, *P* = 0.522	*I*^2^ = 3.8%, *P* = 0.373	*I*^2^ = 66.1%, *P* = 0.032	*I*^2^ = 59.9%, *P* = 0.058	*I*^2^ = 49.6%, *P* = 0.114	*I*^2^ = 50.5%, *P* = 0.109	*I*^2^ = 10.8%, *P* = 0.339
**Region of the Americas**											
The Dominican Republic	1.00 (0.62-1.63)	1.27 (0.54-2.97)	1.92 (0.09-39.72)	11.06 (3.90-31.38)†	1.90 (0.74-4.84)	1.27 (0.54-2.97)	3.27 (1.05-10.19)*	1.05 (0.44-2.48)	5.20 (1.86-14.54)†	0.79 (0.31-2.00)	0.48 (0.18-1.32)
Jamaica	1.06 (0.88-1.28)	0.97 (0.65-1.45)	0.75 (0.29-1.92)	10.70 (6.37-17.97)‡	1.15 (0.66-2.00)	0.97 (0.65-1.45)	1.36 (0.89-2.09)	2.35 (1.49-3.72)†	1.96 (1.31-2.93)†	1.38 (0.86-2.21)	0.53 (0.32-0.88)*
Suriname	1.09 (0.72-1.66)	0.71 (0.32-1.60)	0.83 (0.29-2.37)	21.21 (8.56-52.53)‡	2.21 (0.87-5.59)	0.71 (0.32-1.60)	3.44 (1.48-8.01)†	2.54 (0.94-6.90)	2.60 (0.90-7.52)	0.67 (0.32-1.41)	1.79 (0.81-3.97)
Pooled estimate	1.06 (0.90-1.24)	0.96 (0.69-1.33)	0.82 (0.41-1.63)	12.40 (8.20-18.74)‡	1.46 (0.96-2.23)	0.96 (0.69-1.33)	2.21 (1.10-4.45)*	1.95 (1.19-3.20)†	2.56 (1.48-4.42)†	0.99 (0.61-1.61)	0.77 (0.34-1.73)
	*I*^2^ = 0.0%, *P* = 0.965	*I*^2^ = 0.0%, *P* = 0.620	*I*^2^ = 0.0%, *P* = 0.846	*I*^2^ = 0.0%, *P* = 0.427	*I*^2^ = 0.0%, *P* = 0.411	*I*^2^ = 0.0%, *P* = 0.620	*I*^2^ = 59.7%, *P* = 0.084	*I*^2^ = 29.1%, *P* = 0.244	*I*^2^ = 34.9%, *P* = 0.215	*I*^2^ = 34.5%, *P* = 0.217	*I*^2^ = 71.7%, *P* = 0.029
**Western Pacific**											
Vanuatu	1.00 (0.79-1.27)	0.57 (0.28-1.19)	1.76 (0.76-4.10)	18.89 (8.98-39.73)‡	2.65 (1.03-6.82)*	0.57 (0.28-1.19)	1.17 (0.61-2.23)	4.86 (2.47-9.55)‡	1.67 (0.95-2.94)	0.83 (0.41-1.69)	1.10 (0.49-2.45)
Fiji	1.00 (0.75-1.32)	0.59 (0.33-1.05)	1.29 (0.60-2.78)	16.10 (8.75-29.60)‡	2.42 (1.53-3.81)†	0.59 (0.33-1.05)	3.88 (2.13-7.08)‡	6.06 (3.39-10.83)‡	1.17 (0.69-2.00)	0.69 (0.43-1.11)	1.11 (0.65-1.89)
Wallis and Futuna	0.90 (0.65-1.24)	0.94 (0.31-2.88)	0.92 (0.26-3.18)	7.72 (3.00-19.83)‡	2.46 (0.86-7.07)	0.94 (0.31-2.88)	3.53 (1.54-8.08)†	4.66 (1.47-14.75)*	4.45 (1.71-11.60)†	0.78 (0.32-1.90)	0.53 (0.18-1.51)
Pooled estimate	0.97 (0.83-1.14)	0.62 (0.41-0.95)*	1.37 (0.82-2.29)	14.49 (9.16-22.92)‡	2.46 (1.68-3.61)‡	0.62 (0.41-0.95)*	2.50 (1.13-5.51)*	5.40 (3.58-8.15)‡	1.85 (0.97-3.51)	0.74 (0.52-1.06)	0.99 (0.66-1.50)
	*I*^2^ = 0.0%, *P* = 0.856	*I*^2^ = 0.0%, *P* = 0.735	*I*^2^ = 0.0%, *P* = 0.687	*I*^2^ = 13.4%, *P* = 0.315	*I*^2^ = 0.0%, *P* = 0.986	*I*^2^ = 0.0%, *P* = 0.735	*I*^2^ = 75.0%, *P* = 0.018	*I*^2^ = 0.0%, *P* = 0.857	*I*^2^ = 65.1%, *P* = 0.057	*I*^2^ = 0.0%, *P* = 0.906	*I*^2^ = 0.0%, *P* = 0.456
**Total pooled estimate**	0.96 (0.87-1.06)	0.74 (0.60-0.92)†	1.39 (1.10-1.77)†	12.71 (9.53-16.96)‡	2.39 (2.04-2.80)‡	0.74 (0.60-0.92)†	2.34 (1.79-3.06)‡	4.01 (2.93-5.49)‡	1.82 (1.52-2.18)‡	0.78 (0.66-0.91)†	0.76 (0.61-0.95)†
	*I*^2^ = 62.1%, *P* = 0.001	*I*^2^ = 36.9%, *P* = 0.069	*I*^2^ = 15.7%, *P* = 0.273	*I*^2^ = 61.5%, *P* = 0.001	*I*^2^ = 0.0%, *P* = 0.603	*I*^2^ = 36.9%, *P* = 0.069	*I*^2^ = 55.6%, *P* = 0.004	*I*^2^ = 66.6%, *P* = 0.000	*I*^2^ = 31.4%, *P* = 0.112	*I*^2^ = 6.2%, *P* = 0.383	*I*^2^ = 35.5%, *P* = 0.079

GSHS – Global School-based Student Health Survey, LMICs – low- and middle-income countries, AOR – adjusted odd ratio, CI – confidence interval

*P < 0.05.

†*P* < 0.01.

‡*P* < 0.001.

We found an independent association between sex with multiple partners and cannabis use in 12 out of 16 countries. Regional pooled estimates indicate that the association was significant in all four regions, while between-country heterogeneity was non-significant (*I*^2^ between 0% and 60%). The odds of cannabis use were two to seven times greater in adolescents that had sexual intercourse with two or more people in lifetime than those who had not. School truancy was independently associated with cannabis use in 10 out of 16 countries. Regional pooled estimates indicate that the association was significant in all regions except for the Americas, and between-country heterogeneity was very low (*I*^2^ = 0.0%). Adolescents that missed classes or school without permission on one or more days during the past 30 days had two to four times increased odds of using cannabis. Suicide attempt was associated with cannabis use in nine out of 16 countries. Regional pooled estimates indicate that, the association was statistically significant in all four regions, but between-country heterogeneity was moderate in Southeast Asia (*I*^2^ = 66.1%; *P* = 0.032) and high in Western Pacific (*I*^2^ = 75.0%, *P* = 0.018). Adolescents that attempted suicide during the past 12 months had two to 11 times increased odds of using cannabis during the past 30 days than those who had not.

Although the total pooled associations between cannabis use and physical fighting (adjusted odds ratio (AOR) = 1.82; 95% CI = 1.52-2.18)), perceived school kindness (AOR = 0.78; 95% CI = 0.66-0.91), or parental monitoring (AOR = 0.76; 95% CI = 0.61-0.95) were statistically significant with low between-country heterogeneities, country-specific associations were largely attenuated, as the associations were only evident in less than half or a third of all countries. Similarly, country-specific associations between cannabis use and age, gender, or level of food insecurity were mostly non-existent.

## DISCUSSION

This study is one of the few to examine the association between cannabis and tobacco use in adolescents residing in LMICs. The total pooled prevalence of past 30-day cannabis use in all LMICs examined was 4.3%, slightly higher than the global average (3.8%) in 2015, but lower than that in North America (12.4%), Western and Central Europe (7.2%), and Oceania (10.3%) in 2015 [17]. We found a positive association between past 30-day cannabis and tobacco use across all LMICs, even after controlling for main covariates; tobacco users were over 10 times more likely to engage in cannabis use than non-tobacco users on average. This finding is in line with existing literature reporting a strong association between cannabis and tobacco use generally observed in adolescents residing in more prosperous countries [6,18]. We therefore provide more evidence that use of cannabis and tobacco are strongly intertwined in the adolescent population, possibly regardless of their country’s economic prosperity. Past studies proposed that there might be common genetic influences, environmental risk factors, or common routes of administration for this association, but due to the structure of the GSHS survey, the underlying mechanisms could not be determined in the current study.

The association of cannabis use with covariates is also worth noting. Sex with multiple partners is a common sexual risk behaviour in adolescents and young adults. Many studies found that substance use and sexual risk behaviours tend to cluster in adolescents and young adults [19]. Our study further confirmed these findings; in 12 out of 16 LMICs, adolescents with multiple sex partners had two to seven times increased odds of using cannabis in the past month. Sex with multiple partners plus substance use may further compromise the adolescents’ decision-making process, leading to unprotected sexual behaviours, increasing risks of contracting sexually transmitted infections (STIs) such as human immunodeficiency virus (HIV), and unwanted pregnancies [19,20]. Thus, it has been recommended that sexual health clinics should also routinely offer advice and counselling services on substance use [19]. Considering the high odds of cannabis use among adolescents with multiple sex partners in Asia, a region where such a connection has been rarely explored, customised public health measures are needed to help adolescents with clustering of sexual risk and substance use behaviours.

Although school truancy received less attention in research with adolescents, a few studies have examined its bi-directional relationship with cannabis use, albeit only as their secondary aim [14,21], similar to our study. Some have proposed that truancy may give adolescents unstructured and unsupervised time to socialise with delinquent peers (that used drugs) [21], and that early cannabis use usually occurs in a social context where conventional values such as educational achievement or conformity are rejected, while affiliation with delinquent or substance-using peers is encouraged [22]. Although we could not determine the underlying mechanisms, past-month truancy was associated with two to four times greater odds of past-month cannabis use in 10 out of 16 LMICs. Further studies should examine if interventions aimed at reducing school truancy in adolescents can add help reduce cannabis use.

Cross-sectional studies generally highlighted the importance of the association between cannabis use and suicide attempt, including that in adolescents from LMICs [23-25]. However, there are often several risk factors for suicidal behaviours. Longitudinal studies have reported that substance use, depression, externalising disorders, and family history of drug use explained the association between cannabis use and suicidal behaviours [26,27]. For example, a follow-up study on 1606 adolescents from Québec found the effect of cannabis use on suicidal ideation can be fully explained by substance use (alcohol, tobacco, etc.) [26]. A follow-up study on 3277 adolescents and young adults from the USA found cannabis use no longer predicted suicidal attempt after adjusting for use of alcohol and tobacco, as well as other confounders [27]. Several studies found the causal relationship between cannabis use and suicidal attempt to be gender-dependent [28,29], meaning early cannabis use was associated with later elevated risks of suicide attempt in females, but not in males. We found a cross-sectional association in nine out of 16 instead of all 16 LMICs. Nonetheless, given possible self-harm associated with cannabis use, especially among high-risk groups (i.e. adolescents with suicidal thoughts) [30] and young people residing in regions where cannabis has been legalised for general adult use [31], it should the central focus of drug education/prevention initiatives.

Several meta-analyses found evidence supporting a link between cannabis use and physical violence [32-34], since cannabis use may result in personality change [35,36], psychosis [37], paranoia [38], or impaired executive functions [39], all of which can exacerbate aggression and violence. We found cannabis use was strongly associated with physical fighting in only six out of 16 LMICs. These heterogeneous finding could be attributed to the short timeframe for assessing cannabis use frequency (past 30 days) instead of over a much longer period, or instead of assessing cannabis use potencies.

Parental monitoring is generally considered to be an effective protective factor against delinquent behaviours. Yet, longitudinal studies found parental monitoring wielded a more influential role on cannabis use during early adolescence rather than during young adulthood [40,41]. In the current sample of 12–17-year-old adolescents, parental monitoring was associated with cannabis use in only three out of 16 LMICs. Even though the largely null association may be explained by adjustment for other more relevant covariates of cannabis use, or the assessment of parental monitoring as a “snapshot perception” (past 30 days) rather than an “ongoing process” [40], future studies should examine the role of parental monitoring as a protective factor in the presence of strong risk factors.

### Limitations

This study has several limitations. First, because all inclusion criteria (i.e. LMICs, surveys conducted between 2012-2022, data for all analytic variables included) had to be met, we excluded some LMICs and examined a smaller number of countries than expected, so our findings may not represent each region or all LMICs. Second, the GSHS contains 10 core questionnaire modules, at least six of which have to be included when a country conducts the survey. Although we included most main correlates of cannabis use, some countries did not include modules (i.e. alcohol use) containing important correlates of cannabis use, which might result in overestimation of the strengths of the associations. However, our findings are largely consistent with existing literature. Third, the GSHS predominantly uses single-item rather than multi-item measures of main analytic variables. Measurement theory proposes that multi-item measures are generally more consistent, reliable, and precise [42], so our findings need to be replicated using multi-item scales in prospective studies. Fourth, due to the cross-sectional nature of the GSHS data, we cannot establish causal directions between cannabis use and its significant correlates.

## CONCLUSIONS

Unlike tobacco and alcohol use, cannabis use has not been widely perceived to be associated with adverse consequences other than motor vehicle crashes [33]. Our findings confirmed the strong connection between cannabis and tobacco use found in adolescents from western countries and showed that sexual risk behaviour, suicide attempt, and truancy were also robustly associated with cannabis use in well over half of the LMICs, providing evidence on the clustering of health-risk behaviours in adolescents worldwide. Interventions targeting multiple health risk behaviours with tobacco use as the core behaviour have been associated with more favourable outcomes than those targeting one behaviour only. There is a need to develop comprehensive preventive measures targeting multiple risk behaviours associated with cannabis use for adolescents from LMICs.
